# NRF2 Regulates HER1 Signaling Pathway to Modulate the Sensitivity of Ovarian Cancer Cells to Lapatinib and Erlotinib

**DOI:** 10.1155/2017/1864578

**Published:** 2017-12-19

**Authors:** Ibrahim H. Kankia, Hilal S. Khalil, Simon P. Langdon, Peter R. Moult, James L. Bown, Yusuf Y. Deeni

**Affiliations:** ^1^Division of Science, School of Science Engineering and Technology, Abertay University, Dundee DD1 1HG, UK; ^2^Cancer Research UK Edinburgh Centre and Division of Pathology Laboratory, Institute of Genetics and Molecular Medicine, University of Edinburgh, Crewe Road South, Edinburgh EH4 2XU, UK; ^3^Division of Computing and Mathematics, School of Design and Informatics, Abertay University, Dundee DD1 1HG, UK

## Abstract

NF-E2-related factor 2 (NRF2) regulates the transcription of a battery of metabolic and cytoprotective genes. NRF2 and epidermal growth factor receptors (EGFRs/HERs) are regulators of cellular proliferation and determinants of cancer initiation and progression. NRF2 and HERs confer cancers with resistance to several therapeutic agents. Nevertheless, there is limited understanding of the regulation of HER expression and activation and the link between NRF2 and HER signalling pathways. We show that NRF2 regulates both basal and inducible expression of *HER1*, as treatment of ovarian cancer cells (PEO1, OVCAR3, and SKOV3) with NRF2 activator tBHQ inducing *HER1*, while inhibition of NRF2 by siRNA knockdown or with retinoid represses *HER1*. Furthermore, treatment of cells with tBHQ increased total and phosphorylated NRF2, HER1, and AKT levels and compromised the cytotoxic effect of lapatinib or erlotinib. Treatment with siRNA or retinoid antagonised the effect of tBHQ on NRF2 and HER1 levels and enhanced the sensitivity of ovarian cancer cells to lapatinib or erlotinib. Pharmacological or genetic inhibition of NRF2 and/or treatment with lapatinib or erlotinib elevated cellular ROS and depleted glutathione. This extends the understanding of NRF2 and its regulation of HER family receptors and opens a strategic target for improving cancer therapy.

## 1. Introduction

NF-E2-related factor 2 (NRF2) is a transcription factor that regulates both basal and oxidative stress-induced transcription of many detoxification enzymes and cytoprotective genes, including genes of the metabolic and signal transduction pathways. NRF2 heterodimerizes with small MAF proteins and then binds to *cis*-acting antioxidant response elements (ARE) within the promoters of its target genes to initiate their transcription. However, under normal basal conditions, NRF2 is only freely available at a low level in the cytoplasm with some translocating into the nucleus to regulate the transcription of certain target genes [1, 2].

The epidermal growth factor receptor (EGFR/HER) kinase family is a regulator of cellular proliferation, differentiation, and survival, as well as being factors leading to cancer initiation, maintenance, and progression. HER receptors become active when a soluble ligand binds to their ectodomain, leading to dimerization and subsequent stimulation of the tyrosine kinase, resulting in the phosphorylation of tyrosine residues in the intracellular domain of the receptors. These phosphotyrosine residues serve as docking sites to recruit a number of signal adapter proteins containing SH2 and PTB domains, which link receptor tyrosine kinases (RTKs) to different cellular signalling pathways such as the PI3K/AKT/mTOR, MAPK, and STAT pathways [3–8]. The HER family activation kinetics depend significantly on their expression levels which vary across different cells and cancers. Likewise, it is these variations combined with receptor interactions that drive and confer complexity in the HER receptor family behaviour and function. Overexpression of the HER family has been shown to correlate with poor survival outcomes in women with advanced staged ovarian cancers, who have been treated with cytoreductive surgery and combination therapy [8–13]. HER has also been implicated in ovarian cancer metastases and acting in synergy with various molecular pathways [14–16].

Several studies have implicated NRF2 in promoting resistance to chemotherapeutic agents, in addition to its contribution to general cytoprotection, metabolic reprograming, and cell survival [17–21]. Moreover, targeted chemotherapy involving receptor tyrosine kinase inhibitors (RTKi) against HER family receptors has generated interest as a potential strategy to overcome chemoresistance in breast, ovarian, and other forms of cancers [21–32]. Also, studies have examined the crosstalk between growth-promoting MAPK and PI3K pathways and NRF2 antioxidant pathway in numerous cell systems [1, 33]. However, in the majority of such studies, the focus was on the regulation of NRF2 activity and its function by these kinases. While the interaction and complex formation of NRF2 with HER2 have been reported to enhance HER signalling [22, 24], we recently demonstrated the transcriptional regulation of HER2 and HER3 by NRF2 [22]. Further, we demonstrated a relationship between NRF2 function, HER2/HER3 signalling, ROS generation, and the sensitisation of ovarian cancer cells to the killing effects of the targeted therapeutics, trastuzumab, pertuzumab, or their combination [22, 24].

In this study, we investigated whether NRF2 regulates the expression of HER1/EGFR1, as the findings of such a study may have implications to the success and/or failure of HER-targeted therapies involving RTK inhibitors. We now report on the observed regulation of the HER family receptors by NRF2 to include HER1 (EGFR1). This regulation of HER1 by NRF2 appeared to modulate the sensitivity of a panel of ovarian cancer cells to the RTK inhibitors, lapatinib and erlotinib. This extends our understanding of NRF2 and its regulation of HER family receptors and opens another strategic avenue of manipulating NRF2 to enhance the effectiveness of RTK inhibition in order to kill cancer cells and to overcome resistance to RTKi therapy.

## 2. Materials and Methods

### 2.1. Cell Lines, Culture Conditions, and Treatments

Human ovarian cancer cell lines, PEO1, SKOV3, and OVCAR3, were maintained in RPMI 1640 media (Gibco Invitrogen) supplemented with 10% foetal bovine serum (FBS), 2 mM glutamine, 1 mM sodium pyruvate, 100 *μ*g/mL streptomycin, and 100 U/mL penicillin in an atmosphere of 5% CO_2_ and incubated at 37°C. Before experimental treatments, cells were grown for 24 h in RPMI 1640 media prepared, but replacing FBS, with 5% double charcoal-stripped FBS (Fisher). Heregulin-*β*1 (HRG, Sigma) was used by preparing 1 *μ*mol/L stock solution made with 5% trehalose and 10% FBS in phosphate-buffered saline (PBS) and diluted to a final concentration of 1 nmol/L with media during treatments. Kinase inhibitors targeting HER1 receptor, lapatinib and erlotinib, were used by directly diluting the drugs in media to a final concentration of 5 *μ*M. tert-Butylhydroquinone (tBHQ; Sigma) and bexarotene (Carbosynth) stock solutions were made with dimethylsulfoxide (Fisher) and diluted to a final concentration as required with media. For ROS detection, 2′,7′-dichlorofluorescin diacetate (DCFDA, Sigma) solution was prepared with dimethylsulfoxide in amber tubes to a concentration of 50 mM and stored at −20°C in the dark until used. For the cytotoxicity assay, the CellTiter-Glo® 2.0 assay kit (Promega) was used: stored at −20°C or 4°C in the dark until use.

### 2.2. Reactive Oxygen Species (ROS) Detection

The ROS detection assay was performed with 2′,7′-dichlorofluorescin diacetate (DCFDA) staining (Sigma). Briefly, cells were seeded in triplicate at a density of 0.2 × 10^5^ cells/well in opaque flat bottom 96-well tissue culture plates in 100 *μ*L media without phenol red and allowed to grow for 18 h. Following transfection and/or treatments, cells were washed with PBS and maintained in 100 *μ*L of phenol red-free medium and further incubated for 24 h. A 50 mM stock solution of DCFDA was added to each well containing 100 *μ*L pre-existing media to achieve a final concentration of 25 *μ*M and incubated for 45 min at 37°C. Fluorescence signal intensities indicating ROS levels were recorded by taking readings using a 96-well fluorescent multiplate reader (MODULUS, Promega) using excitation and emission spectra of 485 nm/535 nm. To normalise the fluorescence signal, cells in the same wells were stained with Coomassie brilliant blue stain (Sigma) for 1 h and washed with distilled water and 10% sodium dodecyl sulphate (SDS) solution was added to release the absorbed dye for 10 min while shaking. The absorbance values at 595 nm were then recorded using a multiplate absorbance reader (MODULUS, Promega) and the data was used after normalising the fluorescence values.

### 2.3. Cloning and Expression Vectors Used in the Study

This was as described for the cloning of HER2/HER3 promoters [22, 24]. Briefly, approximately 1.5 kb proximal promoter region of *HER1* was isolated, cloned, and used in the current study. The HER1 primer sequences used for the construct were *HER1* forward: 5′-GTGCTCGAGGCAAGAAGGGTGCATTTTGAAG-3′ and *HER1* reverse: 5′-GTCAAGCTTGTCTCTTGGATGGGCCATC-3′. For the cloning *HER1* promoter (prHER1), total genomic DNA was isolated from human cells using DNeasy Blood and Tissue Kit (Qiagen) and quantified using AstraGene microvolume spectrophotometer (AstraNet). 100 ng of the genomic DNA was used to amplify the *HER1* promoter sequences (MyFi mix, Bioline) using the relevant primers that incorporated *KpnI* and *XhoI* restriction endonuclease sites 5′ and 3′ ends of the amplified promoters. PCR conditions for promoter amplification were initial denaturation of 95°C for 7 min followed by 35 cycles of 95°C for 30 s for denaturation, 50°C for 30 s for annealing, and 72°C for 90 s for extension and a final extension for 10 min at 72°C. The PCR products were run and extracted from agarose gel (Qiagen), digested using *XhoI* and *HindIII* restriction enzymes (Promega), and ligated into PGL3 vector (Promega) to create *HER1* promoter construct (prHER1) driving the expression of luciferase gene for utilisation in dual luciferase reporter assay (Promega). The integrity and authenticity of cloned sequences were determined by sequencing the plasmids using a commercial sequencing service (http://www.dnaseq.co.uk/). All cloned constructs were transfected into relevant cell lines using Lipofectamine 3000 (Life Technologies).

### 2.4. Protein Extraction and Immunoblotting

For immunoblotting, cells were seeded in 60 mm tissue culture plates and grown until 70% confluent. At the time of protein harvest, cells were trypsinized (Gibco Invitrogen) and washed with PBS. Protein lysates were prepared using radioimmune precipitation assay buffer (Pierce Biotech) supplemented with protease and phosphatase inhibitor cocktail (Pierce Biotech) and subjected to sonication of 2 cycles for 10 s at 50% pulse. The final mixture was shaken gently on ice for 15 min, and the proteinous supernatant was obtained following centrifugation of the lysates at 14000*g* for 15 min. Proteins obtained were quantified by Bradford assay (Sigma-Aldrich) using bovine serum albumin as a standard, and sample loading buffer (NuPAGE LDS, Invitrogen) was added to protein lysates, heat denatured at 70°C for 20 min, and stored at −20°C until further use. Prepared protein lysates were loaded into wells of 4–12% gradient SDS-polyacrylamide gels (NuPAGE Bis-Tris gels, Life Technologies) and subjected to electrophoresis at 200 V for 1-2 h. Following this, proteins were transferred onto polyvinylidene difluoride membranes (PVDF, GE Amersham) using the Invitrogen™ iBlot™ 2 Dry Blotting System, a fast western transfer which lasts for only 7 min. Membranes were blocked and then further treated by incubating with relevant primary antibodies (Table 1) for 2 h at room temperature or overnight at 4°C, followed by incubation for 30 min at room temperature with appropriate secondary (anti-rabbit) antibody. Then, following antibody probes, the membranes were processed with Pierce ECL 2 Western blotting substrate (Thermo Scientific) reagent according to manufacturer's protocol. Finally, the membranes and probed proteins were visualised and the images were camera captured using Syngene G-BOX Chemi-XX6 Gel Documentation System (Synoptics, UK). For loading control, immunoblotting of the same lysates was performed and/or reprobed using *β*-actin antibody (Abcam Bioscience, UK).

### 2.5. Luciferase Reporter Assay

For the analysis of promoter activities and transcriptional regulation of *HER1*, the 1.5 kb promoter region of HER1 gene cloned in pGL3 basic vector (Promega) was transfected into relevant cell lines. Briefly, cells (PEO1, OVCAR3, and/or SKOV3) were seeded in triplicate in 24-well plates at a density of 2 × 10^5^ cells per well and allowed to attach for 18 h. The cells were then transfected with either 1 *μ*g of empty pGL3 basic vector (Promega) or pGL3 basic vector with cloned fragments of HER1 promoter driving the expression of luciferase gene, using Lipofectamine 3000 as transfection reagent according to manufacturer's protocol (Life Technologies). Cotransfection was also performed with 0.2 *μ*g of pRL-CMV vector (Promega) to serve as an internal control of transfection and its efficiency. Following this, cells were allowed to grow for 24 h, subjected to desired treatments, and lysed, and the protein lysates were transferred to opaque white bottom 96-well plates for reading. The dual luciferase activities of firefly luciferase (from cloned promoters) and Renilla (internal control) in the harvested lysates were measured sequentially by following manufacturer's protocol (Promega) and taking luminescence readings in a luminometer (MODULUS, Promega). To determine the transcriptional activity of NRF2-dependent ARE promoter in PEO1, OVCAR3, and/or SKOV3 cell lines, basic pGL3 vector (Promega) containing cloned 8× cis-regulatory ARE promoter elements was transfected into the cell lines grown in 24-well plates and also subjected to the dual luciferase reporter assay (Promega).

### 2.6. siRNA Transfection

Small inhibitory RNA (siRNA) was used to genetically knockdown *NRF2* (Hs_NFE2L2_6, Qiagen). For siRNA transfection, cells were seeded in triplicate either in 24-well plates (0.5 × 10^5^ cells), in 60 mm plates with cells grown on poly-L lysine-coated coverslips (0.5 × 10^6^ cells), or in 96-well plates in triplicate (2 × 10 ^4^) and allowed to grow for 24 h. Following this, cells were cotransfected using either 20 pmol siRNA and 1 *μ*g of different PGL3 promoter constructs (24-well plate) or 75 pmol and 100 pmol siRNA only (60 mm plates) or 7 pmol of siRNA (96-well plate) and incubated for further 24 h. Cells transfected in 24-well plates were further processed for dual luciferase assay and those in 60 mm plates were harvested for immunoblotting or used for imaging analysis while those in 96-well plates were processed for cytotoxicity assay. In all cases, scrambled siRNA of appropriate equal quantity to the NRF2-siRNA was used as a control, while transfection was performed using Lipofectamine 3000 (Life Technologies) according to manufacturer's protocol.

### 2.7. Cytotoxicity Assay

The CellTiter-Glo 2.0 assay kit (Promega) was used to evaluate cell viability, as described by the manufacturer. Briefly, cells were seeded in a 96-well plate and allowed to adhere for 18–24 h. Following the duration of treatments of cells with different concentrations of the various compounds, the plate and its contents were equilibrated to room temperature for approximately 30 min. Then, a volume of CellTiter-Glo 2.0 reagent equal to the volume of cell culture medium present in each well was added. The contents were then mixed for 2 min on an orbital shaker to induce cell lysis, and the plate was then incubated at room temperature for 10 min to stabilize the luminescent signal. Finally, the luminescence was recorded using luminometer (MODULUS, Promega). The luminescent signal is proportional to the amount of ATP in the sample, which indicates the presence of living and metabolically active cells.

### 2.8. Measurement of Total Glutathione

The measurement of total glutathione levels was performed using GSH/GSSG-Glo™ Assay kit (Promega) according to manufacturer's protocols as used by [34], and luminescence was recorded using luminometer (MODULUS, Promega).

### 2.9. Statistical Analysis

All statistical analyses were performed using GraphPad Prism software version 6. The significance (value) of differences of pooled results was determined by either independent tests or one-way ANOVA followed by post hoc Tukey's tests. Significance was defined as (^∗^*p* < 0.05, ^∗∗^*p* < 0.01, ^∗∗∗^*p* < 0.001, and ^∗∗∗∗^*p* < 0.0001).

### 2.10. Imaging and Analysis

Images were camera captured using Syngene G-BOX Chemi-XX6 Gel Documentation System (Synoptics, UK). The images were saved in TIFF file format and analysed typically involving the calculation of relative abundance via integrated optical densitometry analysis of each protein band. Densitometry was calculated using ImageJ software and Densitometry 1 Channel plugin (NIH, USA). All values shown are the protein of interest divided by the respective *β*-actin loading control value.

### 2.11. Identification of Putative NRF2 Transcription Sequences

Putative NRF2 transcription sites in the NRF1 promoter region were identified by use of Web-based bioinformatic analysis software [35].

## 3. Results

### 3.1. NRF2 Regulates Both Basal and Inducible Expression of *HER1*

**Figure 1:**
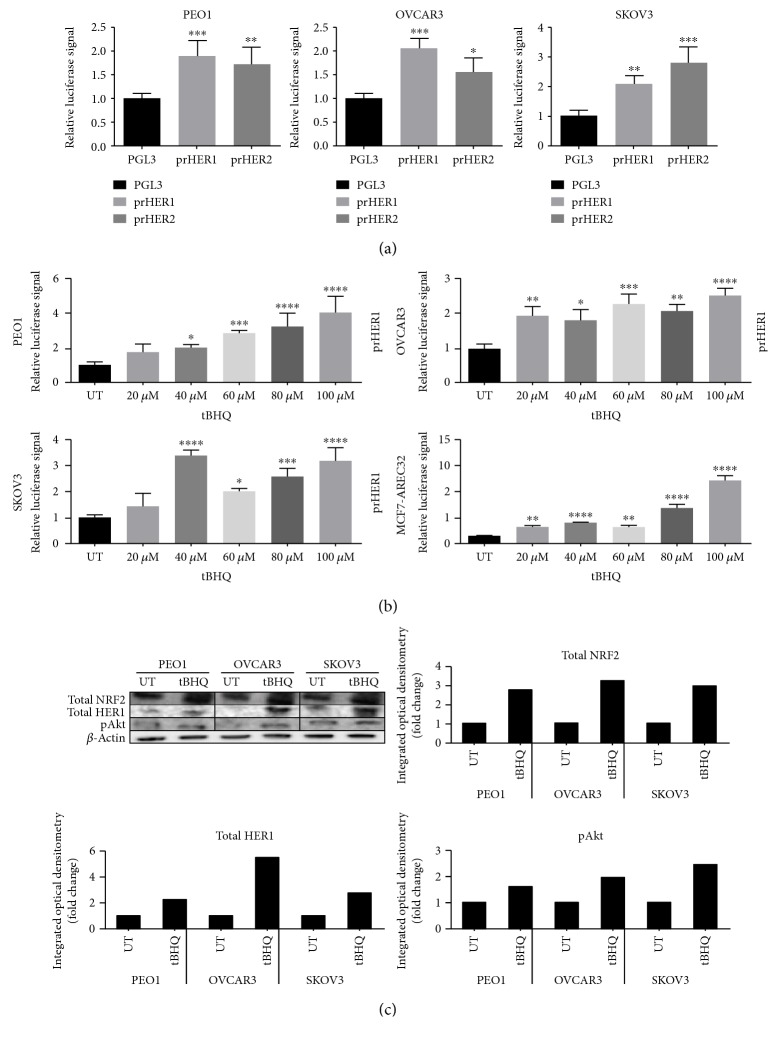
NRF2 regulates both basal and inducible expression of HER1. (a) Cells exhibit different basal expression. Exponentially growing PEO1, SKOV3, and OVCAR3 cells were transfected with either empty PGL3 basic vector or 1 *μ*g PGL3 basic vector with cloned 1.5 kb fragments of either HER1 (prHER1) or HER2 (prHER3) promoter driving the expression of luciferase gene. Cotransfection with 0.2 *μ*g pRL-CMV plasmid was performed as an internal transfection control. (b) tBHQ causes transcriptional induction of HER1 and induction of ARE in a concentration-dependent manner. MCF7-AREc32 which already contains stably cloned 8 × cis-antioxidant response elements (ARE) driving NRF2-dependent expression of luciferase gene was left without any transfection while PEO1, OVCAR3, and SKOV3 cells were transfected with either empty PGL3 basic vector or 1 *μ*g PGL3 basic vector with promoters of HER1-cloned driving HER1 expression of luciferase gene. Cotransfection with 0.2 *μ*g pRL-CMV plasmid was performed as an internal transfection control. Where required PEO1, SKOV3, and OVCAR3 cell lines and MCF7-AREc32 stable cell line were treated in quadruplicate with different concentrations of tBHQ as indicated for 24 h. (c) Immunoblot analysis following treatment with tBHQ demonstrated protein induction of HER1 receptor and also activation of total and with an increase of pAKT. Briefly, exponentially growing cells were either left untreated (UT) or treated with 100 *μ*M tBHQ for 24  h before being harvested and processed for immunoblotting using relevant antibodies. Bar chart showing total NRF2, total HER1, and phospho-Akt levels in PEO1, OVCAR3, and SKOV3 cell lines by quantifying immunoblot signal intensities obtained in the blot image and normalised to the value of UT and expressed as fold change. Data shown in (a) and (b) are the means ± S.D. of triplicates normalised to the value of PGL3 or UT and expressed as fold change with statistical significance determined by one-way ANOVA followed by Tukey's post hoc test (^∗^*p* < 0.05, ^∗∗^*p* < 0.01, ^∗∗∗^*p* < 0.001, and ^∗∗∗∗^*p* < 0.0001).

We have recently demonstrated that NRF2 regulates the expression of drug target HER2 and HER3 family receptors [22]. We now sought to examine whether HER1/EGFR1, another drug target and member of the HER family receptors, is also regulated by NRF2. We used the isolated and cloned HER1 gene-driven luciferase transcriptional reporter construct to examine the basal transcriptional expression of HER1 in a panel of ovarian cancer cell lines (PEO1, OVCAR3, and SKOV3). There were significant high basal level and differential expression of HER1 among the cell lines (Figure 1(a)). We also used similar transcriptional reporter assays to demonstrate basal and differential expression of HER2 receptor which has been shown to be regulated by NRF2 [22].

**Figure 2:**
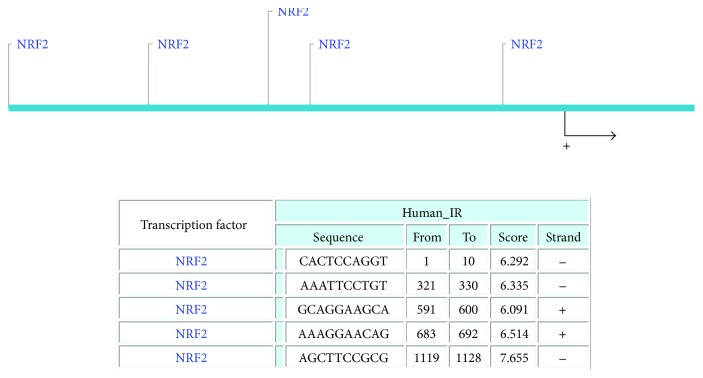
Putative NRF2 binding sites in the promoter region of HER1.

To examine whether NRF2 facilitates the inducible expression of HER1, we repeated the experiment in the presence of *tert*-butylhyroxyquinone (BHQ) a classical activator of NRF2 that acts via AKT [36, 37]. Treatment of cells with varying and increasing concentrations of tBHQ was found to increase the expression of HER1 in all three cell lines (Figure 1(b)). We observed a similar trend and effect of tBHQ on transcriptional control of gene expression following the substitution of the reporter assay and the cell lines with stable clones of MCF7 cells stably expressing 8 × *cis*-elements of antioxidant response (ARE) to drive the expression of luciferase gene (AREc32). To re-evaluate and confirm this observation, cells were treated with either vehicle solvent (controls) or tBHQ (100 *μ*M) for 24 h and total cellular lysates extracted, blotted, and probed for total NRF2, total HER1, and pAKT levels. Pretreatment of cells with tBHQ greatly increased the levels of total NRF2, total HER1, and pAKT (Figure 1(c)). These results demonstrated that activation of NRF2 protein caused upregulation of the HER1/EGFR pathway and activation of total HER1 proteins likely via the PI3K-AKT pathway [38]. This implies that both antioxidant response and EGFR pathways might be subject to coregulatory mechanisms and point to the possible role of NRF2 in mediating the observed transcriptional and translational upregulation of HER1 receptor expression. A search for ARE sites in the promoter region of HER1 identified 5 potential NRF2 binding sites (Figure 2).

### 3.2. Pharmacological and Genetic Inhibition of NRF2 Causes Transcriptional and Translational Downregulation of HER1

**Figure 3:**
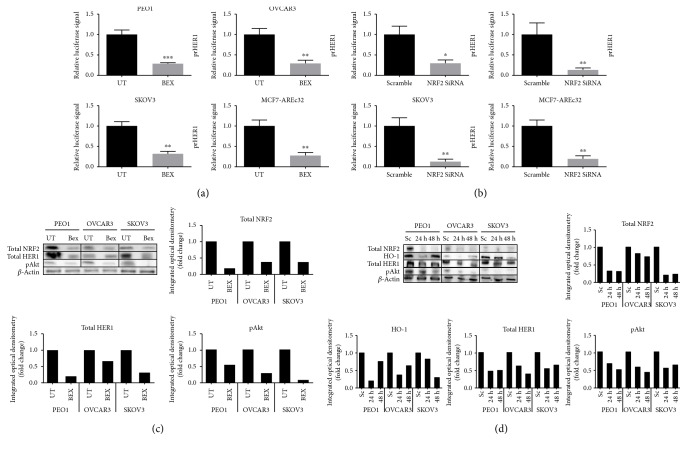
Pharmacological (bexarotene) and genetic inhibition (siRNA) of NRF2 causes transcriptional and translational downregulation of HER1. Luciferase assay showing transcriptional downregulation of HER1 following NRF2 inhibition by (a) bexarotene or (b) siRNA in PEO1, OVCAR3, and SKOV3 cell lines. Exponentially growing PEO1, SKOV3, and OVCAR3 excluding MCF7-AREc32 cell lines were transfected with either empty PGL3 basic vector or 1 *μ*g PGL3 basic vector with cloned HER1 driving the expression of luciferase gene. Cotransfection with 0.2 *μ*g pRL-CMV plasmid was performed as an internal transfection control as described in the Materials and Methods. At 24 h posttransfection, cells were (a) either left untreated or treated with 2.5 *μ*M bexarotene. (b) Cells were either transfected with scrambled siRNA (Sc) or transfected with 20 pmol of NRF2 siRNA (Si) for 24 h. Following treatments, lysates were prepared and luciferase activity was measured using dual luciferase reporter assay (Promega) in multiplate reader (MODULUS, Promega). (c) Immunoblot analysis following treatment with bexarotene demonstrated protein downregulation of HER1 receptor and decrease of NRF2, HO-1, and HER1. Exponentially growing cells were either left untreated (UT) or treated with 2.5 *μ*M bexarotene for 24 h before being harvested and processed for immunoblotting using relevant antibodies. Bar chart showing total NRF2, HO-1, and total HER1 levels in PEO1, OVCAR3, and SKOV3 cell lines by quantifying immunoblot signal intensities obtained in (c). (d) Immunoblot analysis following knockdown of NRF2 demonstrated protein downregulation of both HER1 receptor and decrease of NRF2 and HO-1 in PEO1, OVCAR3, and SKOV3 cell lines. Cells were either transfected with scrambled siRNA (Sc) or transfected with 75 pmol of NRF2 siRNA (Si). After 24 h and 48 h, cells were harvested and processed for immunoblotting using relevant antibodies. *β*-Actin of the same blot was used as loading control. Bar chart shows the levels of relevant proteins by quantifying immunoblot signal intensities obtained and expressed as fold change. Data shown in (a) and (b) are the means ± S.D. of triplicates, normalised to UT or scramble expressed in fold change with statistical significance determined by Student's *t*-test (^∗^*p* < 0.05, ^∗∗^*p* < 0.01, and ^∗∗∗^*p* < 0.001).

To further delineate the role of NRF2 in the regulation of HER1 receptor expression, we next sought to examine the expression of HER1 receptor following the antagonism of NRF2 and its function. Previous studies have used retinoid or siRNA to pharmacologically or genetically inhibit NRF2 and its function [20, 22, 24, 39]. Thus, we next set up to pharmacologically or genetically inhibit NRF2 by examining ovarian cancer cells treated with either retinoid/rexinoid bexarotene or an NRF2 specific/targeting siRNA (Figure 3). Our developed *HER1* gene-driven luciferase reporter system and Western blot analysis of HER1 and NRF2 levels were used to evaluate and delineate the role of NRF2 in the regulation of HER1 receptor expression. In all three cell lines tested, the basal transcription levels of *HER1* (Figures 3(a) and 3(b)) and the basal levels of total HER1 and total NRF2 and pAKT (Figures 3(c) and 3(d)) were repressed by both bexarotene and siRNA. Repressed levels of total HO-1 (Figure 3(d)), a classical NRF2-regulated cytoprotective gene, was also observed. A similar trend on transcriptional repression of the control of gene expression by either bexarotene or siRNA was also observed following the substitution of the reporter assay and the cell lines with the stable clones of MCF7 AREc32 cells stably expressing luciferase gene under the control of 8 × *cis*-elements of antioxidant response (ARE) to which NRF2 is commonly known to bind to as *trans*-acting transcription factor.

**Figure 4:**
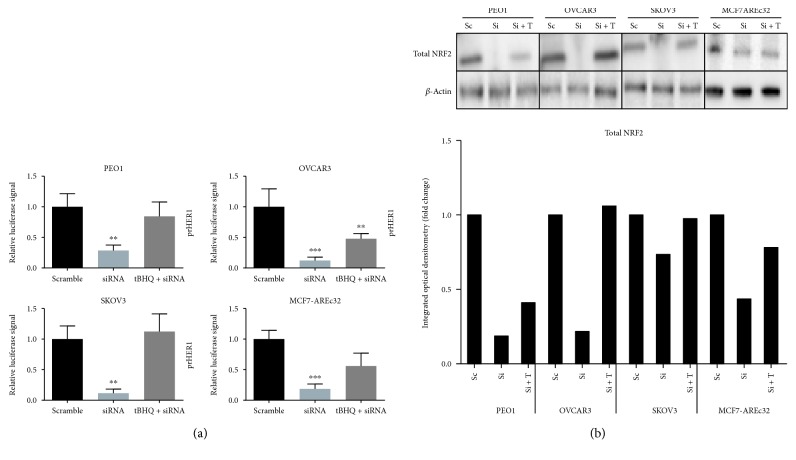
Treatment with tBHQ reduces the knockdown effect of siRNA. (a) siRNA-mediated knockdown of NRF2 causes inhibition of its transcriptional antioxidant program and repression of HER1 level in both constitutive and tBHQ-induced states. MCF7-AREc32 which already contains stably cloned 8× *cis*-antioxidant response elements (ARE) driving NRF2-dependent expression of luciferase gene was left without any transfection while PEO1, OVCAR3, and SKOV3 cells were transfected with either empty PGL3 basic vector or 1 *μ*g PGL3 basic vector with promoters of HER1-cloned driving HER1 expression of luciferase gene. Cotransfection with 0.2 *μ*g pRL-CMV plasmid was performed as an internal transfection control. Where required, cotransfection with either scrambled RNA (Sc) or NRF2 siRNA was performed using 20 pmol siRNA. At 24 h after transfection, treatment with 100 *μ*M tBHQ was performed where indicated for 4 h following which cells were processed for dual luciferase reporter assay (Promega) to record luciferase activity in multiplate reader (MODULUS, Promega). (b) Immunoblotting analysis showing repression of NRF2 following NRF2 knockdown by siRNA in PEO1, OVCAR3, and SKOV3 cell lines. Cells were either transfected with scrambled siRNA (Sc) or transfected with 75 pmol of NRF2 siRNA (Si). After 48 h, cells were either left untreated or treated with 100 *μ*M tBHQ (T) for 4 h, before being processed for immunoblotting using relevant antibodies. *β*-Actin of the same blot was used as loading control. Bar chart shows NRF2 levels by quantifying immunoblot signal intensities obtained and expressed as fold change. Data in (a) are the means with ±S.D. of triplicates, normalised to scramble with statistical significance determined by one-way ANOVA followed by Tukey's post hoc test. ^∗∗^*p* < 0.01 and ^∗∗∗^*p* < 0.001.

Further evidence to implicate NRF2 in the regulation of HER1 expression was obtained when cotreatment of the ovarian cancer and MCF7 AREc32 cells with tBHQ and NRF2-siRNA compromised the tBHQ-dependent induction of either HER1 promoter-driven or ARE-driven luciferase gene expression in the ovarian cancer cells or in MCF7 AREc32 cells (Figure 4(a)), respectively. This inhibitory effect of siRNA on the tBHQ-dependent induction of gene expression (Figure 4a) appeared to be concomitant with decreased total NRF2 levels in all the cells tested (Figure 4(b)), with albeit a marginal reduction of total NRF2 in the OVCAR3 cell line.

These findings collectively strengthen and support the role of NRF2 in mediating the observed downregulation of *HER1* expression, at both transcriptional and translational levels, following the treatment of cells with either bexarotene or siRNA against NRF2.

### 3.3. Pharmacological or Genetic Inhibition of NRF2 by Bexarotene or siRNA Elevates Cellular Reactive Oxygen Species (ROS) and Depletes Glutathione (GSH) Levels

**Figure 5:**
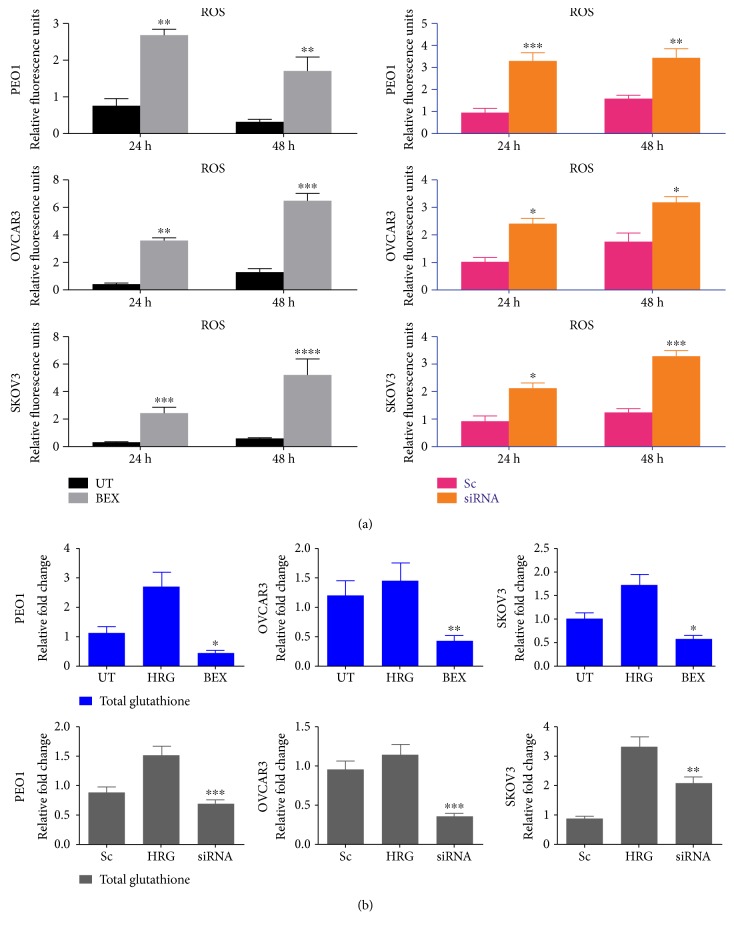
Inhibition and knockdown of NRF2 by bexarotene and siRNA, respectively, elevate the level of ROS and depletion of total glutathione level. (a) Bexarotene treatment and knockdown of NRF2 by siRNA cause increase in ROS levels. Exponentially growing cells were seeded in triplicates in opaque flat bottom black-walled 96-well plates for 24 h. Following this, cells were either left untreated (UT) or treated with 2.5 *μ*M bexarotene or 7 pmol of siRNA (scrambled or targeted) for different time points as indicated. Following incubations, cells were loaded with DCFDA fluorescent stain for 45 min and assayed for ROS by measuring fluorescence as described in Materials and Methods. (b) Bexarotene and siRNA cause depletion of total glutathione. Exponentially growing cells were seeded in 60 mm tissue culture plates for 24 h and either left untreated (UT) or treated with media containing 1 nM heregulin alone (HRG) or with cotreatment of 2.5 *μ*M bexarotene or 100 pmol siRNA for 24 h before being harvested to prepare protein lysates and processed for glutathione assay as described in Materials and Methods. Data is shown as fold change of bexarotene or siRNA-treated cells to UT or scrambled siRNA, respectively, with statistical significance determined by one-way ANOVA followed by Tukey's post hoc test. ^∗^*p* < 0.05, ^∗∗^*p* < 0.01, ^∗∗∗^*p* < 0.001, and ^∗∗∗∗^*p* < 0.0001.

Reactive oxygen species (ROS) are recognized as second messengers in signal transduction processes and cytoprotection by influencing growth, survival, and overall physiological homeostasis [40, 41]. Also, biochemical strategies to curb ROS include nonenzymatic and low molecular weight scavengers, such as glutathione (GSH), and enzymatic antioxidant defense systems that include GSH biosynthetic enzymes, superoxide dismutases, catalases, peroxidases, thioredoxins, peroxiredoxins, and reductases [42, 43] Most importantly, NRF2 drives both the basal and inducible transcription of genes associated with redox homeostasis and cytoprotection, as well as other signal transduction pathways [44]. Therefore, to further examine the relationship between NRF2, ROS, and the regulation of HER1, we next quantified total basal ROS following NRF2 inhibition and knockdown to determine whether NRF2 depletion caused elevation of ROS. Loading of cells with 2′,7′-dichlorofluorescin diacetate dye, which is a fluorescent marker of intracellular ROS, following pretreatment with either bexarotene or siRNA confirmed the presence and elevation of ROS for 24 and 48 h (Figure 5(a)), as resultant consequences from both NRF2 inhibition and siRNA-dependent knockdown (Figure 5(a)).

Following the observed inhibition of NRF2 and increase in ROS and downregulation of HER1 receptor expression and levels in ovarian cancer cells after their treatment with bexarotene and siRNAs, we anticipated that NRF2 knockdown with siRNA and/or treatment with bexarotene would cause a depletion of total cellular GSH. To examine this, cells were either left untreated or treated with bexarotene or siRNA. Cells stimulated with heregulin (HRG) which is also reported to induce cellular glutathione were used as a positive control. After 24 h treatment, HRG induced total cellular GSH, while both bexarotene and siRNA caused significant depletion of GSH levels in all cells (Figure 5(b)). This study demonstrates that perturbation in cellular redox status can influence the expression of HER1 receptor and may have implications for HER1 receptor-targeted therapies.

### 3.4. Chemotherapeutic and Mechanism of Action of RTK Inhibitors, Lapatinib and Erlotinib, Involves Generation of ROS and GSH Depletion in Ovarian Cancer

**Figure 6:**
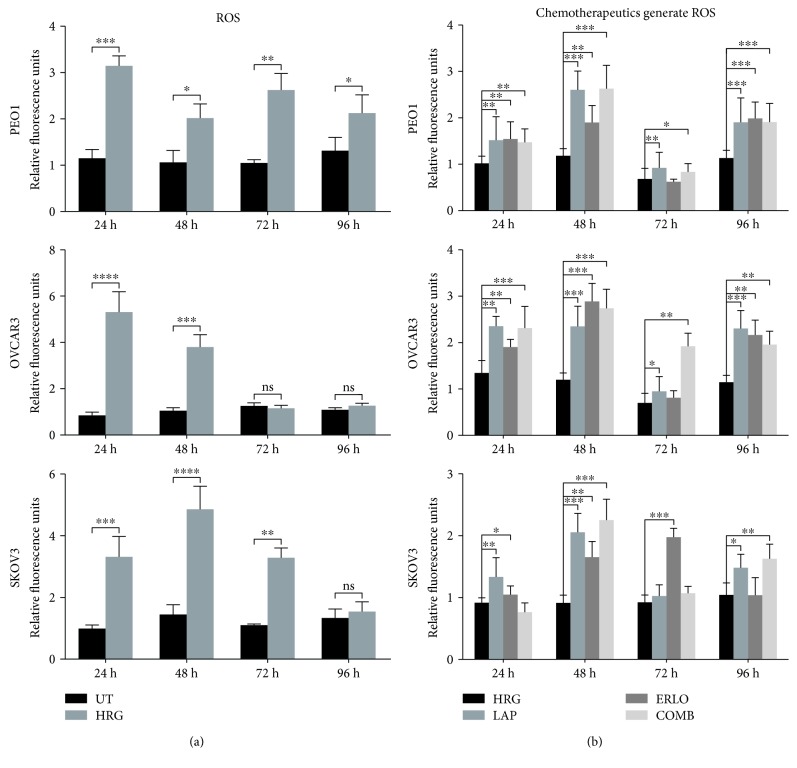
Treatment with lapatinib and erlotinib generates reactive oxygen species (ROS) in ovarian cancer cells. (a) Heregulin treatment causes persistent elevation of ROS in ovarian cancer cells. Exponentially growing cells were seeded in triplicates in opaque flat bottom black-walled 96-well plates for 24 h. Following this, cells were either left untreated (UT) or treated with 1 nM heregulin for different time points as indicated. Following incubations, cells were loaded with DCFDA fluorescent stain for 45 min and assayed for ROS as described in Materials and Methods. (b) Lapatinib, erlotinib, and their combination cause ROS generation. Cells were seeded as in (a) and treated with either 1 nM HRG alone or with cotreatment of 5 *μ*M lapatinib (LAP), erlotinib (ERLO), or their combination (COMB) for different time points as indicated, and ROS assay was repeated. For both (a) and (b), the fluorescence reading recorded from each well was normalised to total cell abundance within the same wells as described in Materials and Methods. Data shown are mean values ± S.D of triplicates, normalised to UT in (a) or HRG in (b) and expressed as fold change. Statistical significance was determined between treatment groups either by independent *t*-test or one-way ANOVA followed by post hoc Tukey's test as appropriate and significance expressed according to the scale: (^∗^*p* < 0.05, ^∗∗^*p* < 0.01, ^∗∗∗^*p* < 0.001, and ^∗∗∗∗^*p* < 0.0001).

We have recently demonstrated the connection between NRF2 status and the modulation of HER2/HER3 family receptors, ROS, and the mechanism of action and effectiveness of targeted immunotherapy against ovarian cancer cells [22, 24]. In this current study, we have also shown similar connectivity of NRF2 levels, HER1 modulation, and ROS levels in ovarian cancer cells. These findings suggested that while retinoids/rexinoids, like retinoic acid (RA) and bexarotene, inhibit the NRF2-dependent AR pathway, such treatment might also elevate cellular ROS levels in the ovarian cancer cell lines. This led us to hypothesize that the cytotoxic action of RTK inhibition targeting HER1 receptor (lapatinib and erlotinib) involves cellular accumulation of ROS concomitant to the disruption of NRF2 and its function. To address this hypothesis using lapatinib and erlotinib, firstly total ROS levels in basal, HRG stimulated, and drug-inhibited states in all three cell lines were studied. Here as well, HRG which is known to be a potent ligand for HER receptors was used. The data in (Figure 6(a)) illustrated that HRG stimulation alone led to a significant increase in ROS levels in all three cell lines as compared to basal levels in unstimulated cells. Moreover, it is seen that treatment with lapatinib, erlotinib, or their combination led to ROS generation in all the ovarian cancer cell line models. ROS elevation was seen at all the time points (24, 48, 72, and 96 h) tested, with observed elevation of ROS being differential in cell- and time-dependent fashion. For example, there was more significant elevation of ROS in PEO1 cells (at later time points) as compared to OVCAR3 and SKOV3 (Figure 6(a)).

Investigation of the single drug treatment (lapatinib or erlotinib), in all the cell lines (Figure 6(b)), showed that lapatinib often generated more ROS than erlotinib, while their combination failed to generate higher level of ROS than their singular administration at all the time points investigated. However, the fact that administration of these drugs led to generation of ROS (Figure 6(b)) suggests that ROS could be a contributing factor in cellular cytotoxicity of lapatinib and erlotinib and implicates the engagement of AR pathway and inhibition of NRF2 function during drug action. Thus, we next sought to investigate the status of the NRF2-ARE antioxidant response of cells following lapatinib and erlotinib treatments.

### 3.5. Lapatinib and Erlotinib Disrupt Antioxidant Transcriptional Response, Suppress NRF2 and HO-1 Protein Levels, and Elevate Cellular ROS

**Figure 7:**
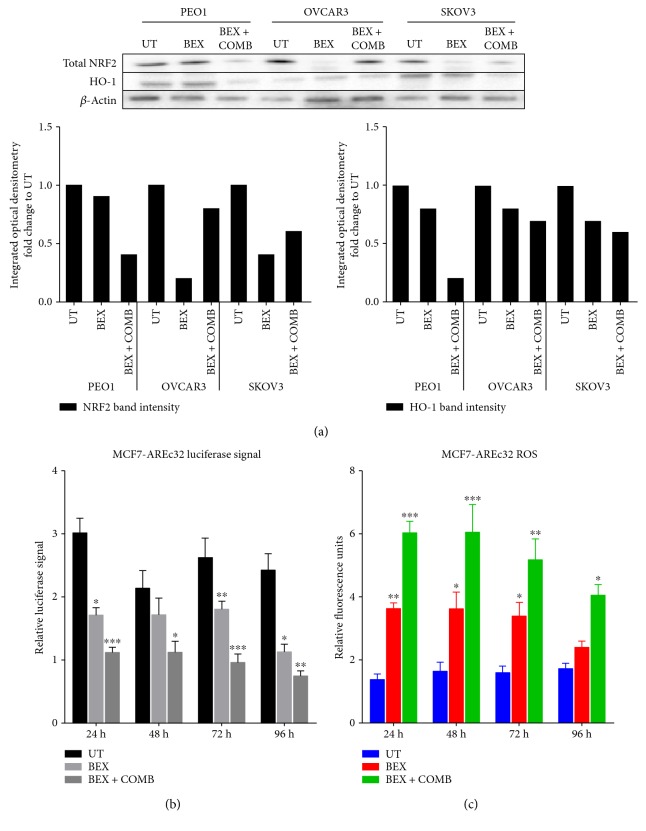
Treatment with bexarotene causes inhibition of NRF2-dependent antioxidant response pathway and generates ROS. (A) Western analysis showing repression of NRF2 and HO-1 levels following bexarotene treatment in PEO1, OVCAR3, and SKOV3 cell lines. Exponentially growing cells were either left untreated, treated with 2.5 *μ*M bexarotene, or a combination of 2.5 *μ*M bexarotene together with 5 *μ*M of lapatinib and erlotinib for 24 h before being harvested to prepare protein lysates and processed as described in Materials and Methods. *β*-Actin was used as loading control. The bars indicate NRF2 and HO-1 levels following quantification of immunoblot signal intensities obtained in (a) and normalised to the value of UT and expressed as fold change. The signal intensities of bands were quantified through integrated optical densitometry measurement. (b) Bexarotene treatment causes inhibition of NRF2-dependent transcription. Exponentially growing AREc32 cell line stably expressing 8× *cis*-antioxidant response elements driving the expression of luciferase gene in an NRF2-dependent manner were either left untreated (UT), treated with bexarotene alone, or with bexarotene and combination of lapatinib and erlotinib for different time points as indicated. Following this, cell lysates were prepared and assayed for luciferase activity (BrightGlo Luciferase System, Promega). (c) Bexarotene treatment causes increase in ROS levels. Exponentially growing AREc32 cell lines stably expressing 8× *cis*-antioxidant response elements driving the expression of luciferase gene in an NRF2-dependent manner were seeded in triplicates in opaque flat bottom black-walled 96-well plates for 24 h. Following this, cells were either left untreated (UT), treated with bexarotene alone, or with bexarotene and combination of lapatinib or erlotinib for different time points as indicated. Following incubations, cells were loaded with DCFDA fluorescent stain for 45 min and assayed for ROS as described in Materials and Methods. Data are the mean values ± S.D of quadruplicates, normalised to untreated (UT) and expressed as fold change with statistical significance determined by one-way ANOVA followed by Tukey's post hoc test according to the scale ^∗^*p* < 0.05, ^∗∗^*p* < 0.01, ^∗∗∗^*p* < 0.001.

Bexarotene which on its own is reported to be an anticancer agent has previously been shown to inhibit NRF2/ARE in an NRF2-dependent manner [39]. In order to extend the observations reported in the previous section, the consequences of NRF2-ARE inhibition following exposure to the chemotherapeutic drugs were investigated. Experiments were performed, firstly in MCF7-AREc32 cell line and subsequently in the ovarian cancer cell line models in order to validate and confirm the inhibitory action of bexarotene and also drugs (lapatinib and erlotinib) on the NRF2-dependent AR pathway. Exposure to bexarotene alone caused a decrease in total NRF2 levels in OVCA3 and SKOV3 cells (Figure 7a). Interestingly, the levels of NRF2 in these cell lines were further decreased following cotreatment with combined chemotherapy (lapatinib and erlotinib) in PE01 cells. The drug-induced reduction in NRF2 levels suggests that chemotherapy is also targeting NRF2. Next, using the luciferase ARE reporter MCF7-AREc32 cell line, it was found that bexarotene treatment significantly inhibited transcriptional activity of NRF2 at all the time points tested (Figure 7(b)). Bexarotene treatment of MCF7-AREc32 reporter cell line also elevated ROS levels (Figure 7(c)). Furthermore, bexarotene enhanced the inhibitory action of the combination of lapatinib and erlotinib on the AR pathway. Also, bexarotene alone and in combination with lapatinib and erlotinib reduced the level of HO-1 (Figure 7(a)). These findings suggested that while bexarotene inhibits NRF2-dependent AR pathway, such treatment might also elevate cellular ROS levels in the ovarian cancer cell lines. Indeed, treatment with bexarotene significantly represses total NRF2 and induced ROS in all three cell lines (Figure 5(a)).

### 3.6. Activity of Lapatinib, Erlotinib, and Bexarotene Involves Repression of NRF2-Dependent Transcription and Depletion of Total Glutathione

**Figure 8:**
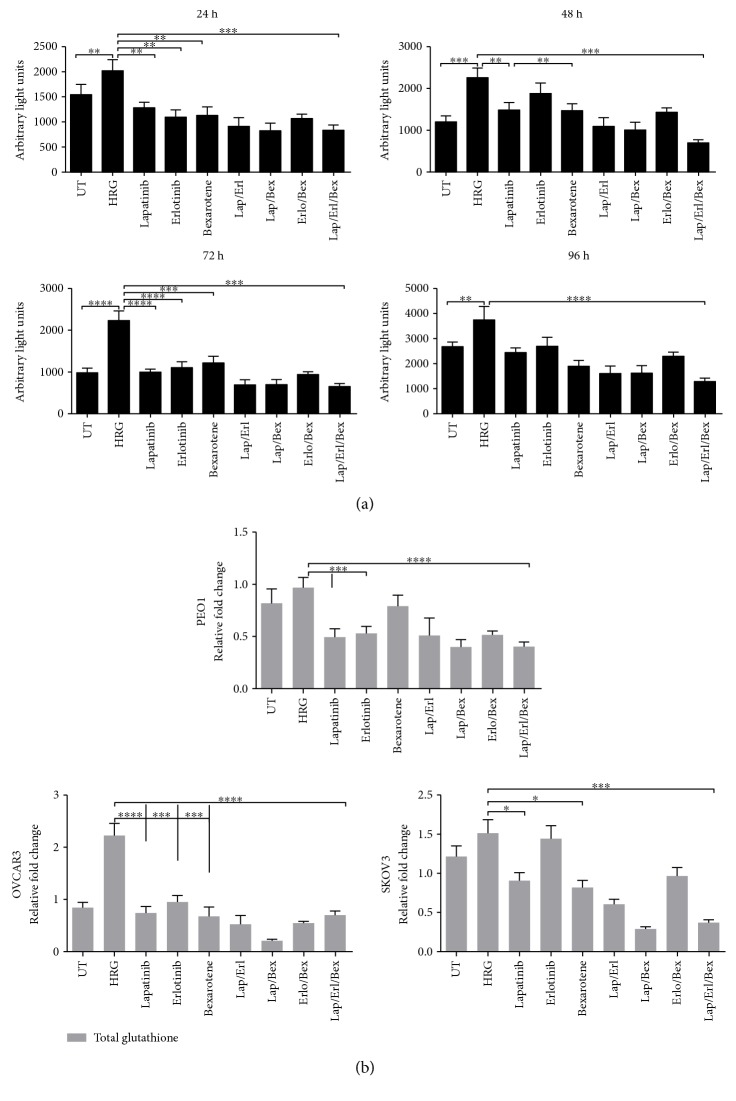
Treatment with lapatinib, erlotinib, and bexarotene causes inhibition of NRF2-dependent transcription and depletion of total glutathione levels. (a) Single and combination of lapatinib and erlotinib cause inhibition of NRF2-dependent transcription. Exponentially growing MCF7-AREc32 cell lines stably expressing *cis*-regulatory antioxidant response elements driving the expression of luciferase gene in an NRF2-dependent manner were treated with 1 nM HRG alone or with cotreatment of 5 *μ*M lapatinib and erlotinib either individually or in combination for different time points as indicated. Following this, cell lysates were prepared and assayed for luciferase activity as described in Materials and Methods. Data shown are mean values ± S.D of quadruplicates, normalised to untreated (UT) and expressed as fold change with statistical significance determined by one-way ANOVA followed by Tukey's post hoc test. Asterisks indicate significant differences between individual groups as indicated and according to the scale ^∗^*p* < 0.05, ^∗∗^*p* < 0.01, and ^∗∗∗^*p* < 0.001. (b) Single and combination of lapatinib and erlotinib cause decrease in glutathione level. Exponentially growing cells were seeded in 60 mm tissue culture plates for 24 h and either left untreated (UT) or treated with media containing 1 nM heregulin alone (HRG) or with cotreatment of 5 *μ*M lapatinib and erlotinib or their combination with 2.5 *μ*M bexarotene (COMB) for 72 h before being harvested to prepare protein lysates and processed for glutathione assay. Data are mean values ± S.D of triplicates and expressed as fold change to the UT. Statistical significance was determined by one-way ANOVA followed by Tukey's post hoc test according to the scale ^∗^*p* < 0.05, ^∗∗^*p* < 0.01, ^∗∗∗^*p* < 0.001, ^∗∗∗∗^*p* < 0.0001.

Based on the additional decreased levels of NRF2 observed in the MCF7-AREC32 stable cell line following combined lapatinib and erlotinib with bexarotene (Figure 7(a)), it appeared that single treatment with lapatinib or erlotinib or combination could also inhibit NRF2/ARE-dependent transcription. The MCF7-AREc32 cell line as a luciferase reporter was used. The results (Figure 8(a)) show that with HRG stimulation a potent ligand for HER receptors only, a significant induction of AR pathway was observed. However, the cotreatment with combination of lapatinib and erlotinib and with single drug alone disrupted and suppressed the ARE-dependent induction significantly thereby inhibiting NRF2 function. Combination of either lapatinib and bexarotene or erlotinib and bexarotene also disrupted the function of NRF2, and the greatest inhibition of NRF2/ARE activity was observed when the cells were treated with a combination of lapatinib, erlotinib, and bexarotene (Figure 8(a)).

We envisaged these different treatments of cells (Figure 8(a)) to lead to perturbations in cellular levels of total GSH, as previously some of these treatments have resulted in elevated ROS (Figures 5(a), 6, and 7(c)) and GSH depletion (Figure 5(b)) in cells. Thus, to investigate whether NRF2 repression would also lead to depletion of total cellular GSH, the panel of ovarian cancer cells (PEO1, OVCAR3, and SKOV3) was treated in the same manner as the AREc32 cells (Figure 8(a)) for 96 h. It was found that the 96 h treatment of ovarian cancer cells with HRG elevated total cellular GSH levels, while combination of HRG with lapatinib, erlotinib, or bexarotene significantly reduced the GSH levels in all the cell lines, albeit with most reduction observed in PEO1 (Figure 8(b)). Generally, the combination of lapatinib with erlotinib, lapatinib with bexarotene, or erlotinib with bexarotene caused more significant GSH depletion than singular treatments. Contrary to our initial expectation, treatment of cells with treble drug combination (lapatinib, erlotinib, and bexarotene) did not cause further GSH depletion than the combination of any two drugs. These results indicated that NRF2 inhibition, ROS accumulation, and GSH depletion may be contributing to the unique mechanism of cytotoxicity of lapatinib or erlotinib and that bexarotene enhances the mechanism of action and the cytotoxicity of lapatinib and/or erlotinib. Overall, this provides support and strengthens the hypothesis that the cellular cytotoxicity of lapatinib and/or erlotinib involves the engagement of the AR pathway and the concomitant inhibition of NRF2 function during drug action.

### 3.7. NRF2 Inhibition Sensitises EGFR Pathway to HER1 Targeting Agents

The observation that retinoid (bexarotene) or tBHQ treatment caused downregulation or upregulation of HER1 at transcriptional and protein levels suggests that NRF2 may be directly involved in regulating HER receptor expression and as such might have a role in responses to targeted chemotherapies involving HER1. The cellular cytotoxicity of HER targeting lapatinib and/or erlotinib culminates with the engagement of AR pathway and the concomitant inhibition of NRF2 function during drug action.

**Figure 9:**
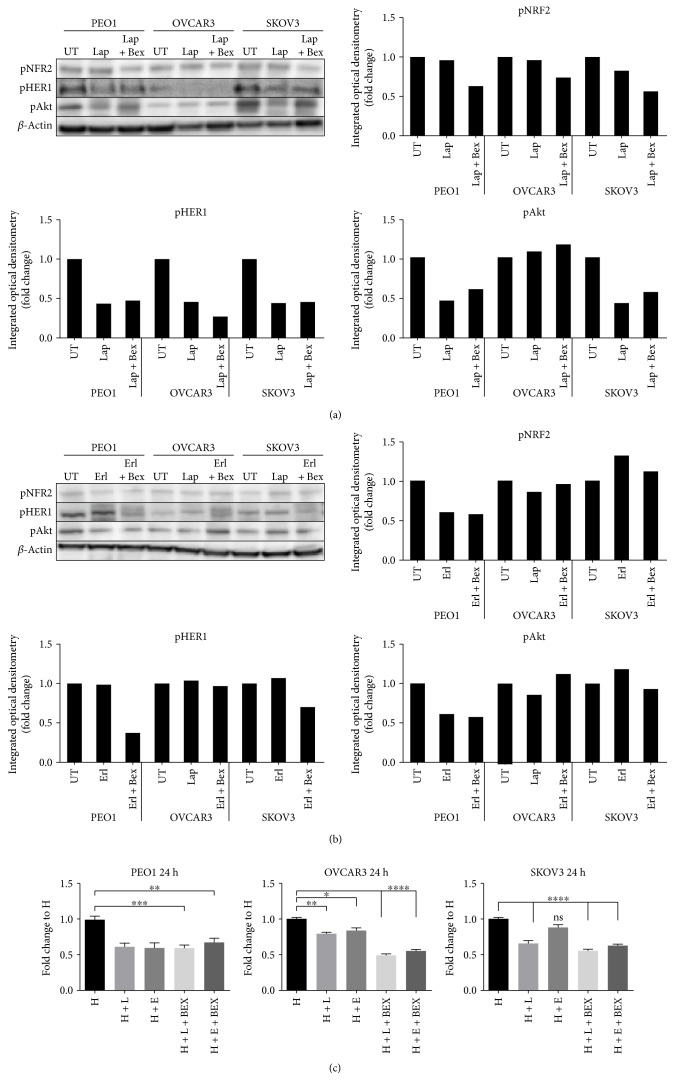
NRF2 inhibition with bexarotene sensitises EGFR signalling pathway to HER1 inhibitors (a) lapatinib and (b) erlotinib. Immunoblot analysis showing bexarotene-dependent repression of EGFR signalling following its combination with lapatinib and erlotinib. Exponentially growing cells were either left untreated in media containing 1 nM heregulin (UT) or treated with the same media containing in the presence of 1 nM heregulin with lapatinib (Lap) alone or erlotinib (Erl) alone, each at 5 *μ*M, or with cotreatment of 2.5 *μ*M bexarotene (Lap + Bex) or (Erl + Bex) for 24 h before and processed for immunoblotting using relevant antibodies, and *β*-actin was used as loading control. (c) NRF2 knockdown and inhibition increase the chance of cytotoxicity of HER family-targeted agents, lapatinib and erlotinib in ovarian cancer cells. Exponentially growing cells were seeded in triplicates in 96-well plates for 24 h. Following this, cells were either left untreated in media containing 1 nM heregulin (H) or treated with same media containing in the presence of 1 nM HRG with 5 *μ*M each of lapatinib (H + L) or erlotinib (H + E) or treated with combination 5 *μ*M lapatinib and 2.5 *μ*M bexarotene (H + L + BEX) or combination of 5 *μ*M erlotinib and 2.5 *μ*M bexarotene (H + E + BEX). Cell number was assessed indirectly by use of the cell titre glo assay. Data shown are means ± S.D. of triplicates, normalised to (H), expressed in fold change with statistical significance was calculated by one-way ANOVA followed by Tukey's post hoc test according to the scale: (^∗^*p* < 0.05, ^∗∗^*p* < 0.01, ^∗∗∗^*p* < 0.001, and ^∗∗∗∗^*p* < 0.0001).

To answer this important question, PEO1, OVCAR3, and SKOV3 cells either with lapatinib and erlotinib alone or by cotreatment with retinoid/rexinoid (bexarotene) were investigated to examine the consequences of NRF2 inhibition on drug effects and cellular responses. Treatment with lapatinib alone represses both pNRF2 and pHER1 levels in the cell lines (Figures 9(a) and 9(b)). There was also similar concomitant marked repression of pAKT levels in PEO1 and SKOV cell lines by either drug and in combination with bexarotene. These results are consistent with lapatinib or erlotinib inhibiting NRF2 activity and repressing HER1/EGFR. Moreover, cotreatment with an NRF2 inhibitor (bexarotene) can further repress the EGFR signalling pathway and might sensitize the ovarian cancer cells to the killing effects of lapatinib and/or erlotinib. Furthermore, data in Figure 9(c) appears to lend support to these assertions, as we observed significant increased cytotoxicity of lapatinib or erlotinib following the pharmacological inhibition of NRF2 with bexarotene for 24 h in OVCAR3 and SKOV3. We failed to record any such increased cytotoxicity of lapatinib or erlotinib in PEO1 with the addition of bexarotene; however, PEO1 appeared to be more sensitive to the cytotoxic effects of lapatinib or erlotinib.

## 4. Discussion

HER1 is a member of the EGFR kinase family which is a driver of cellular proliferation, differentiation, and survival [45–47]. This investigation has identified the transcriptional (Figure 1(b)) and translational (Figure 1(c)) regulatory roles of NRF2 for HER1 receptor. We first demonstrated that NRF2 activation by tBHQ not only induced the NRF2 dependent antioxidant response (AR) pathway as expected, but interestingly also increased HER1 protein levels (Figure 1(c)). tBHQ is widely known as an NRF2 activator and can upregulate ARE response-driven genes. Several possible NRF2 binding sites were identified in the HER1 promoter region (Figure 2). The regulatory role of NRF2 on HER1 expression is supported by our observed higher basal levels of HER1 protein in PEO1 and SKOV3 then in OVCAR3 (Figures 1(b) and 3(b)), as PEO1 and SKOV3 have been shown before [48] to have higher basal levels of NRF2 than in OVCAR3. On the other hand, pharmacological (bexarotene) and genetic inhibition of NRF2 downregulated the basal expression (Figures 3(a) and 3(b)) and levels of HER1 in the cells (Figure 3). Previous studies have shown the inhibitory nature of siRNA and retinoids on AR element [20, 22, 49, 50] and we have used similar approaches to demonstrate that NRF2 regulates HER2 and HER3 [22]. Interestingly, the regulation of HER1 by NRF2 appeared to involve AKT (Figures 1(c), 3(c), 3(d), 9(a), and 9(b)), and the HER family receptor heterodimers are known to be very powerful elicitors of the PI3K/AKT/mTOR pathway [51, 52]. Furthermore, elements of the receptor-regulated PI3K and MAPK are known to regulate NRF2 function [52, 53], while several features of RTK signalling are regulated by ROS which are invariably controlled by NRF2 function [37, 54, 55]. Thus, the HER receptor and AR pathways do share common substrates, and both pathways are cytoprotective and prosurvival in nature, in addition to both being implicated in anticancer drug resistance. Therefore, the regulation of HER family receptors, in addition to HER2 and HER3, is currently extended by this report to include HER1 receptor and has implications for the anticancer effectiveness of receptor tyrosine kinase inhibitors (RTKi) like lapatinib and erlotinib that target HER1 receptor.

The small molecule cancer drugs lapatinib and erlotinib have been in clinical use over the last decade [56]. They are novel oral dual tyrosine kinase inhibitors blocking HER1 and HER2 pathways that present beneficial effects on breast and lung cancers with positive HER2. They are also recognized as promising therapeutics targeting the increased EGFR expression in triple-negative breast cancer (TNBC), specifically through their use with other chemotherapies [57–60]. However, the efficacy of these drugs is potentially reduced due to limited therapeutic efficacy and frequent emergence of resistance. The resistance to these molecularly targeted agents can be due to mutation of the target itself, as in the case of kinase gatekeeper mutations, the activation of adaptive feedback loops, or alternative oncogenic pathways [60–63]. It is interesting that in most cases the resistance mechanism preserves the original overall pathway addiction, for example, to the RAS-RAF-MEK-ERK or PI3 kinase-AKT kinase signal transduction cascades. However, the rational design of combinations to overcome such problems, as well as the issue of clonal heterogeneity, still proves challenging [56, 64, 65].

The curative activity of retinoids in the treatment of patients with acute promyelocytic leukaemia harbouring translocations in the *RARα* receptor gene established the validity of the concept of targeting pathogenetic driver abnormalities with a small molecule in the clinic [56, 64]. Bexarotene is a retinoid/rexinoid reported to be chemopreventive and chemotherapeutic agents [20, 66, 67], which like retinoic acid (RA) regulates cell proliferation, differentiation, and morphogenesis. It inhibits tumorigenesis through suppression of cell growth and stimulation of cellular differentiation [67, 68]. Bexarotene is an effective oral retinoid therapy for the treatment of early and advanced-stage cutaneous T-cell lymphoma (CTCL), especially in patients who have failed on other therapies [69, 70]. Interestingly, this work has highlighted the usefulness of bexarotene to inhibit NRF2 function, to produce ROS, to deplete GSH, and to modulate the expression and levels of HER1 in ovarian cancer cells. We showed that NRF2 is likely to be involved in regulating the transcriptional and translational expression of HER1 receptor and as such might have a role in responses to targeted chemotherapies involving HER1. Furthermore, the cellular cytotoxicity of HER targeting lapatinib and/or erlotinib appeared to involve the engagement and perturbation of AKT and AR pathways leading to the inhibition of NRF2 function during drug action in all the cell lines (Figures 9(a) and 9(b)). There was also similar concomitant marked repression of pAKT levels by either drug and in combination with bexarotene. These results demonstrated that lapatinib or erlotinib inhibits NRF2 activity to repress HER1/EGFR, and also, bexarotene, an NRF2 inhibitor, can further repress EGFR signalling pathway to further sensitize and enhance the killing effects of lapatinib and/or erlotinib against OVCAR3 and SKOV3 ovarian cancer cells (Figures 9(c)). Although lapatinib and/or erlotinib appeared to be more cytotoxic towards PEO1 than against OVCAR3 and/or SKOV3 cells, we could not record any increased cytotoxicity of lapatinib or erlotinib with bexarotene in PEO1. This may be related to PEO1's propensity and addiction to very high levels and greatly nuclear localised NRF2 [48]; however, PEO1 appeared to be more sensitive to the cytotoxic effects of lapatinib or erlotinib. These results indicated that NRF2 inhibition, downregulation of HER1 and AKT expression (in particular pHER1, pAKT, and pNRF2), ROS accumulation, and GSH depletion inform the basis and the unique mechanism of cytotoxicity of lapatinib or erlotinib and that bexarotene enhances the mechanism of action and the cytotoxicity of lapatinib and/or erlotinib.

Overall, this supports and strengthens the hypothesis that the cellular cytotoxicity of lapatinib and/or erlotinib involves the engagement of AR pathway and the concomitant inhibition of NRF2 function during drug action. In addition to this study, the foregoing hypothesis is also supported by the reports that pharmacological inhibition of PI3K/mTOR inhibition is critical for achieving optimal response to lapatinib [57, 61, 71, 72]. Furthermore, HER2 requires HIF-1 for tumour growth and that HIF is a major downstream regulator of HER2 that protects cells from anoikis and metabolic stress caused by decreased matrix adhesion [73]. Moreover, hypoxia/HIF1*α* induces lapatinib resistance in HER2-positive breast cancer cells via regulation of DUSP2 and HIF-1 can bypass the lapatinib-treated inhibition of the ERK pathway via inhibition of the dual-specificity phosphatase 2 (DUSP2). Since it is well known that several genes and components of the PI3K/AKT/mTOR, MAPK, and HIF-1 pathways, as well as HER receptors, are under NRF2-driven transcriptional and functional control [22, 74, 75], then, there is novelty in inhibiting NRF2 function to augment the mechanism of action and effectiveness of anticancer and RTK targeting therapeutics like lapatinib and erlotinib. Moreover, a combinatorial targeted therapy, lapatinib, and/or erlotinib plus bexarotene may effectively overcome lapatinib and/or erlotinib resistance in vivo and could be further tested in preclinical and clinical trials for ovarian and other cancer types. Also, the consideration and evaluation of NRF2 as biomarker of susceptibility and/or resistance to RTK inhibition is an attractive and a timely proposal.

## 5. Conclusion

This study has demonstrated that perturbation in cellular redox status can influence the expression of HER1 receptor and with implication for HER1 receptor-targeted therapies. These data suggest that NRF2 regulates HER1 and that combined treatment of bexarotene, the NRF2 inhibitor, with distinct HER1 inhibitory agents (lapatinib and/or erlotinib) can augment the potency of HER1 and RTK signalling inhibition. The findings in this research have opened up a new potential avenue of improving the effectiveness of lapatinib and erlotinib when combined with bexarotene for the treatment of ovarian cancer. The present study offered new insights into a novel molecular mechanism of action and effectiveness of lapatinib and/or erlotinib and identified NRF2 as an important potential target for treatment of lapatinib/erlotinib-resistant cancers.

## Figures and Tables

**Table 1:** Antibodies used in the study.

		Catalogue	
Antibody	Host	Number	Company
HER1-EP38Y	Rabbit	ab52894	Abcam
pHER1	Rabbit	ab40815	Abcam
NRF2	Rabbit	ab89443	Abcam
pNRF2-EP1809Y	Rabbit	ab76026	Abcam
pAkt-Ser473	Rabbit	ab9271	Cell signalling
HO-1	Rabbit	5853S	Cell signalling
HRP-linked anti-secondary antibody	Rabbit	7074	Cell signalling
*β*-Actin	Rabbit	ab1801	Abcam

## References

[1] Namani A., Li Y., Wang X. J., Tang X. (2014). Modulation of NRF2 signaling pathway by nuclear receptors: implications for cancer. *Biochimica et Biophysica Acta (BBA) - Molecular Cell Research*.

[2] Tsuchida K., Tsujita T., Hayashi M. (2017). Halofuginone enhances the chemo-sensitivity of cancer cells by suppressing NRF2 accumulation. *Free Radical Biology and Medicine*.

[3] Yarden Y. (2001). The EGFR family and its ligands in human cancer: signalling mechanisms and therapeutic opportunities. *European Journal of Cancer*.

[4] Normanno N., De Luca A., Bianco C. (2006). Epidermal growth factor receptor (EGFR) signaling in cancer. *Gene*.

[5] Yarden Y., Sliwkowski M. X. (2001). Untangling the ErbB signalling network. *Nature Reviews Molecular Cell Biology*.

[6] Gschwind A., Fischer O. M., Ullrich A. (2004). The discovery of receptor tyrosine kinases: targets for cancer therapy. *Nature Reviews Cancer*.

[7] Ritter C. A., Arteaga C. L. (2003). The epidermal growth factor receptor–tyrosine kinase: a promising therapeutic target in solid tumors. *Seminars in Oncology*.

[8] Marmor M. D., Skaria K. B., Yarden Y. (2004). Signal transduction and oncogenesis by ErbB/HER receptors. *International Journal of Radiation Oncology Biology Physics*.

[9] Bianco R., Melisi D., Ciardiello F., Tortora G. (2006). Key cancer cell signal transduction pathways as therapeutic targets. *European Journal of Cancer*.

[10] Friedlander M. L. (1998). Prognostic factors in ovarian cancer. *Seminars in Oncology*.

[11] Psyrri A., Kassar M., Yu Z. (2005). Effect of epidermal growth factor receptor expression level on survival in patients with epithelial ovarian cancer. *Clinical Cancer Research*.

[12] Bull Phelps S. L., Schorge J. O., Peyton M. J. (2008). Implications of EGFR inhibition in ovarian cancer cell proliferation. *Gynecologic Oncology*.

[13] Ledermann J. A., Raja F. A. (2010). Targeted trials in ovarian cancer. *Gynecologic Oncology*.

[14] Alečković M., Kang Y. (2015). Regulation of cancer metastasis by cell-free miRNAs. *Biochimica et Biophysica Acta (BBA) - Reviews on Cancer*.

[15] Clayton A. J., Danson S., Jolly S. (2004). Incidence of cerebral metastases in patients treated with trastuzumab for metastatic breast cancer. *British Journal of Cancer*.

[16] Hough C. D., Sherman-Baust C. A., Pizer E. S. (2000). Large-scale serial analysis of gene expression reveals genes differentially expressed in ovarian cancer. *Cancer Research*.

[17] Hayes J. D., McMahon M. (2009). NRF2 and KEAP1 mutations: permanent activation of an adaptive response in cancer. *Trends in Biochemical Sciences*.

[18] Hayes J. D., Ashford M. L. J. (2012). Nrf2 orchestrates fuel partitioning for cell proliferation. *Cell Metabolism*.

[19] Hayes A. J., Skouras C., Haugk B., Charnley R. M. (2015). Keap1–Nrf2 signalling in pancreatic cancer. *The International Journal of Biochemistry & Cell Biology*.

[20] Wang X. J., Hayes J. D., Henderson C. J., Wolf C. R. (2007). Identification of retinoic acid as an inhibitor of transcription factor Nrf2 through activation of retinoic acid receptor alpha. *Proceedings of the National Academy of Sciences of the United States of America*.

[21] Tang X., Wang H., Fan L. (2011). Luteolin inhibits Nrf2 leading to negative regulation of the Nrf2/ARE pathway and sensitization of human lung carcinoma A549 cells to therapeutic drugs. *Free Radical Biology and Medicine*.

[22] Khalil H. S., Langdon S. P., Kankia I. H., Bown J., Deeni Y. Y. (2016). NRF2 regulates HER2 and HER3 signaling pathway to modulate sensitivity to targeted immunotherapies. *Oxidative Medicine and Cellular Longevity*.

[23] Goltsov A., Deeni Y., Khalil H. S. (2014). Systems analysis of drug-induced receptor tyrosine kinase reprogramming following targeted mono- and combination anti-cancer therapy. *Cell*.

[24] Khalil H. S., Langdon S. P., Goltsov A. (2016). A novel mechanism of action of HER2 targeted immunotherapy is explained by inhibition of NRF2 function in ovarian cancer cells. *Oncotarget*.

[25] Langdon S. P., Lawrie S. S., Hay F. G. (1988). Characterization and properties of nine human ovarian adenocarcinoma cell lines. *Cancer Research*.

[26] Langdon S. P., Faratian D., Nagumo Y., Mullen P., Harrison D. J. (2010). Pertuzumab for the treatment of ovarian cancer. *Expert Opinion on Biological Therapy*.

[27] Langdon S. P., Cameron D. A. (2013). Pertuzumab for the treatment of metastatic breast cancer. *Expert Review of Anticancer Therapy*.

[28] Mullen P., Cameron D. A., Hasmann M., Smyth J. F., Langdon S. P. (2007). Sensitivity to pertuzumab (2C4) in ovarian cancer models: cross-talk with estrogen receptor signalling. *Molecular Cancer Therapeutics*.

[29] Tu S. H., Ho C. T., Liu M. F. (2013). Luteolin sensitises drug-resistant human breast cancer cells to tamoxifen via the inhibition of cyclin E2 expression. *Food Chemistry*.

[30] Messersmith W. A., Ahnen D. J. (2008). Targeting EGFR in colorectal cancer. *The New England Journal of Medicine*.

[31] Paez J. G., Jänne P. A., Lee J. C. (2004). EGFR mutations in lung cancer: correlation with clinical response to gefitinib therapy. *Science*.

[32] Regales L., Gong Y., Shen R. (2009). Dual targeting of EGFR can overcome a major drug resistance mutation in mouse models of EGFR mutant lung cancer. *Journal of Clinical Investigation*.

[33] Ahmed S. M. U., Luo L., Namani A., Wang X. J., Tang X. (2017). Nrf2 signaling pathway: pivotal roles in inflammation. *Biochimica et Biophysica Acta (BBA) - Molecular Basis of Disease*.

[34] Granata A., Nicoletti R., Perego P. (2015). Global metabolic profile identifies choline kinase alpha as a key regulator of glutathione-dependent antioxidant cell defense in ovarian carcinoma. *Oncotarget*.

[35] Sandelin A., Wasserman W. W., Lenhard B. (2004). ConSite: web-based prediction of regulatory elements using cross-species comparison. *Nucleic Acids Research*.

[36] Wang L., Chen Y., Sternberg P., Cai J. (2008). Essential roles of the PI3 kinase/Akt pathway in regulating Nrf2-dependent antioxidant functions in the RPE. *Investigative Ophthalmology and Visual Science*.

[37] Bryan H. K., Olayanju A., Goldring C. E., Park B. K. (2013). The Nrf2 cell defence pathway: Keap1-dependent and -independent mechanisms of regulation. *Biochemical Pharmacology*.

[38] Reddy N. M., Potteti H. R., Vegiraju S., Chen H.-J., Tamatam C. M., Reddy S. P. (2015). PI3K-AKT signaling via Nrf2 protects against hyperoxia-induced acute lung injury, but promotes inflammation post-injury independent of Nrf2 in mice. *PLoS One*.

[39] Wu J., Wang H., Tang X. (2014). Rexinoid inhibits Nrf2-mediated transcription through retinoid X receptor alpha. *Biochemical and Biophysical Research Communications*.

[40] Forman H. J., Maiorino M., Ursini F. (2010). Signaling functions of reactive oxygen species. *Biochemistry*.

[41] Mittler R., Vanderauwera S., Suzuki N. (2011). ROS signaling: the new wave?. *Trends in Plant Science*.

[42] Nordberg J., Arnér E. S. J. (2011). Reactive oxygen species, antioxidants, and the mammalian thioredoxin system. *Free Radical Biology and Medicine*.

[43] Sies H., Sharov V. S., Klotz L. O., Briviba K. (1997). Glutathione peroxidase protects against peroxynitrite-mediated oxidations a new function for selenoproteins as peroxynitrite reductase. *Journal of Biological Chemistry*.

[44] Jaiswal A. K. (2004). Nrf2 signaling in coordinated activation of antioxidant gene expression. *Free Radical Biology and Medicine*.

[45] Cao C., Lu S., Sowa A. (2008). Priming with EGFR tyrosine kinase inhibitor and EGF sensitizes ovarian cancer cells to respond to chemotherapeutical drugs. *Cancer Letters*.

[46] Gu D. M., Lu P. H., Zhang K. (2015). EGFR mediates astragaloside IV-induced Nrf2 activation to protect cortical neurons against in vitro ischemia/reperfusion damages. *Biochemical and Biophysical Research Communications*.

[47] Gui T., Shen K. (2012). The epidermal growth factor receptor as a therapeutic target in epithelial ovarian cancer. *Cancer Epidemiology*.

[48] Khalil H. S., Goltsov A., Langdon S. P., Harrison D. J., Bown J., Deeni Y. (2015). Quantitative analysis of NRF2 pathway reveals key elements of the regulatory circuits underlying antioxidant response and proliferation of ovarian cancer cells. *Journal of Biotechnology*.

[49] McMahon M., Campbell K. H., MacLeod A. K., McLaughlin L. A., Henderson C. J., Wolf C. R. (2014). HDAC inhibitors increase NRF2-signaling in tumour cells and blunt the efficacy of co-adminstered cytotoxic agents. *PLoS One*.

[50] Wang B., Zhu X., Kim Y. (2012). Histone deacetylase inhibition activates transcription factor Nrf2 and protects against cerebral ischemic damage. *Free Radical Biology and Medicine*.

[51] Garrett J. T., Olivares M. G., Rinehart C. (2011). Transcriptional and posttranslational up-regulation of HER3 (ErbB3) compensates for inhibition of the HER2 tyrosine kinase. *Proceedings of the National Academy of Sciences*.

[52] Zipper L. M., Mulcahy R. T. (2000). Inhibition of ERK and p38 MAP kinases inhibits binding of Nrf2 and induction of GCS genes. *Biochemical and Biophysical Research Communications*.

[53] Kang K. W., Ryu J. H., Kim S. G. (2000). The essential role of phosphatidylinositol 3-kinase and of p38 mitogen-activated protein kinase activation in the antioxidant response element-mediated rGSTA2 induction by decreased glutathione in H4IIE hepatoma cells. *Molecular Pharmacology*.

[54] Aslan M., Özben T. (2003). Oxidants in receptor tyrosine kinase signal transduction pathways. *Antioxidants and Redox Signaling*.

[55] Nguyen T., Nioi P., Pickett C. B. (2009). The Nrf2-antioxidant response element signaling pathway and its activation by oxidative stress. *Journal of Biological Chemistry*.

[56] Hoelder S., Clarke P. A., Workman P. (2012). Discovery of small molecule cancer drugs: successes, challenges and opportunities. *Molecular Oncology*.

[57] Brady S. W., Zhang J., Seok D., Wang H., Yu D. (2014). Enhanced PI3K p110α signaling confers acquired lapatinib resistance that can be effectively reversed by a p110α-selective PI3K inhibitor. *Molecular Cancer Therapeutics*.

[58] Karakashev S. V., Reginato M. J. (2015). Hypoxia/HIF1 induces lapatinib resistance in ERBB2-positive breast cancer cells via regulation of DUSP2. *Oncotarget*.

[59] El Guerrab A., Bamdad M., Bignon Y. J., Penault-Llorca F., Aubel C. (2017). Anti-EGFR monoclonal antibodies enhance sensitivity to DNA-damaging agents in BRCA1-mutated and PTEN-wild-type triple-negative breast cancer cells. *Molecular Carcinogenesis*.

[60] Scrima M., Zito Marino F., Oliveira D. M. (2017). Aberrant signaling through the HER2-ERK1/2 pathway is predictive of reduced disease-free and overall survival in early stage non-small cell lung cancer (NSCLC) patients. *Journal of Cancer*.

[61] Nakagawa T., Takeuchi S., Yamada T. (2012). Combined therapy with mutant-selective EGFR inhibitor and Met kinase inhibitor for overcoming erlotinib resistance in EGFR-mutant lung cancer. *Molecular Cancer Therapeutics*.

[62] Lee C. K., Wu Y. L., Ding P. N. (2015). Impact of specific epidermal growth factor receptor (EGFR) mutations and clinical characteristics on outcomes after treatment with EGFR tyrosine kinase inhibitors versus chemotherapy in EGFR-mutant lung cancer: a meta-analysis. *Journal of Clinical Oncology*.

[63] Rosell R., Carcereny E., Gervais R. (2012). Erlotinib versus standard chemotherapy as first-line treatment for European patients with advanced EGFR mutation-positive non-small-cell lung cancer (EURTAC): a multicentre, open-label, randomised phase 3 trial. *Lancet Oncology*.

[64] Jeon W. K., Hong H. Y., Kim B. C. (2011). Genipin up-regulates heme oxygenase-1 via PI3-kinase-JNK1/2-Nrf2 signaling pathway to enhance the anti-inflammatory capacity in RAW264.7 macrophages. *Archives of Biochemistry and Biophysics*.

[65] Rodriguez K. J., Wong H. K., Oddos T., Southall M., Frei B., Kaur S. (2013). A purified feverfew extract protects from oxidative damage by inducing DNA repair in skin cells via a PI3-kinase-dependent Nrf2/ARE pathway. *Journal of Dermatological Science*.

[66] Tan K. P., Kosuge K., Yang M., Ito S. (2008). NRF2 as a determinant of cellular resistance in retinoic acid cytotoxicity. *Free Radical Biology and Medicine*.

[67] Garattini E., Bolis M., Garattini S. K. (2014). Retinoids and breast cancer: from basic studies to the clinic and back again. *Cancer Treatment Reviews*.

[68] Mongan N. P., Gudas L. J. (2007). Diverse actions of retinoid receptors in cancer prevention and treatment. *Differentiation*.

[69] Assaf C., Bagot M., Dummer R. (2006). Minimizing adverse side-effects of oral bexarotene in cutaneous T-cell lymphoma: an expert opinion. *British Journal of Dermatology*.

[70] Scarisbrick J. J., Morris S., Azurdia R. (2013). UK consensus statement on safe clinical prescribing of bexarotene for patients with cutaneous T-cell lymphoma. *British Journal of Dermatology*.

[71] Gayle S. S., Arnold S. L., O'Regan R. M., Nahta R. (2012). Pharmacologic inhibition of mTOR improves lapatinib sensitivity in HER2-overexpressing breast cancer cells with primary trastuzumab resistance. *Anti-Cancer Agents in Medicinal Chemistry*.

[72] O'Brien N. A., McDonald K., Tong L. (2014). Targeting PI3K/mTOR overcomes resistance to HER2-targeted therapy independent of feedback activation of AKT. *Clinical Cancer Research*.

[73] Whelan K. A., Schwab L. P., Karakashev S. V. (2013). The oncogene HER2/neu (ERBB2) requires the hypoxia-inducible factor HIF-1 for mammary tumor growth and anoikis resistance. *Journal of Biological Chemistry*.

[74] Malhotra D., Portales-Casamar E., Singh A. (2010). Global mapping of binding sites for Nrf2 identifies novel targets in cell survival response through ChIP-Seq profiling and network analysis. *Nucleic Acids Research*.

[75] Hirotsu Y., Katsuoka F., Funayama R. (2012). Nrf2–MafG heterodimers contribute globally to antioxidant and metabolic networks. *Nucleic Acids Research*.

